# Use of Sport Supplements and Doping Substances by Athletes: Prevalence and Relationships

**DOI:** 10.3390/jcm13237132

**Published:** 2024-11-25

**Authors:** Philip Hurst, Maria Kavussanu, Rachael Davies, Neil Dallaway, Christopher Ring

**Affiliations:** 1 School of Psychology and Life Sciences, Canterbury Christ Church University, Canterbury CT1 1QU, UK; 2 School of Sport, Exercise and Rehabilitation Sciences, University of Birmingham, Birmingham B15 2TT, UK

**Keywords:** beliefs, doping, ergogenic aids, supplements

## Abstract

**Background**: The use of sport supplements may represent a risk factor for the use of doping in sports. To explore this putative risk, the current study examined the frequency of sport supplement use and associations between the use of sport supplements and the use of doping substances and methods in athletes. **Methods**: The participants (*n* = 345; 56% male, 22 ± 5 years, 18–43 years) completed measures of sport supplement use, sport supplement beliefs, doping likelihood, and doping use. Based on the Australian Institute of Sport’s “Sports Supplement Framework”, the participants were asked whether they used 23 sport supplements from four categories (sport foods, medical supplements, performance supplements, other). They were also asked whether they used six classes of doping substances and methods (alphabodies, stimulants, steroids, erythropoietin, growth hormone, blood doping). **Results**: Sport supplements were used by 96% of the athletes, whereas doping substances were used by 4% of the athletes. Moreover, athletes who used more sport supplements also used more doping substances. The use of sport supplements was related to the use of doping substances both directly and indirectly via sport supplement beliefs and doping likelihood. **Conclusions**: Consumption of sport supplements is common, whereas doping is rare, and, moreover, the belief that sport supplements help optimize performance in competitive sport confers an increased risk for doping.

## 1. Introduction

Sport supplements, defined as “a food, food component, nutrient, or non-food compound that is purposefully ingested in addition to the habitually consumed diet with the aim of achieving a specific health and/or performance benefit” [1], are permitted by sport organizations [2], and widely used by athletes [3,4]. Athletes use sport supplements for a variety of reasons, including energy provision, health maintenance, management of nutrient deficiencies, recovery from injury, and performance enhancement [1,5,6,7,8].

The use of sport supplements has been linked with doping (i.e., the use of banned substances and methods). A meta-analysis showed that athletes who used sport supplements were 2.74 times more likely to dope and reported greater intentions and more favorable attitudes to dope than non-users [9]. Cross-sectional studies typically find that doping prevalence [10,11,12] and willingness [7] are higher among athletes who identify as supplement users than non-users. However, only a small fraction of athletes who use sport supplements also use doping substances and methods [11,13,14]. For instance, Hurst et al. (2023) [9] reported that 86.3% of those who used sport supplements did not dope. Thus, while sport supplements may increase the likelihood of an athlete doping, only a small proportion of sport supplement users go on to dope. This funneling effect has encouraged researchers to search for reasons why some athletes progress from using sport supplements to doping substances and methods.

The gateway hypothesis [15] has been used to explain the supplement–doping relationship. This hypothesis, first developed to explain recreational drug use, proposes a sequential progression from the use of “soft” drugs, such as tobacco or alcohol, to the use of “hard” drugs, such as marijuana and cocaine [15]. In the context of doping, athletes who use permitted substances, such as sport supplements, might be expected to progress to using prohibited substances, such as steroids and stimulants [10]. In line with this hypothesis, a retrospective study of students found that the use of protein preceded the use of creatine, which, in turn, preceded the use of steroids, which were used by 5.8% of the sample [16].

The gateway hypothesis considers the development of beliefs that the consumption of substances, including sport supplements, benefits athletic performance. In support of this notion, a qualitative study of elite cyclists reported that if athletes took a substance (sport supplement) in a race and won, they would take that substance in every race thereafter [17]. Therefore, the development of strong beliefs about the effectiveness of performance-enhancing substances could be a mechanism that leads users of supplements to become users of doping substances. Indeed, beliefs about the effectiveness of sport supplements may create beliefs that prohibited substances are more effective for performance enhancement, thereby placing such athletes at increased risk for doping [18].

Qualitative research reveals that athletes who believe sport supplements are necessary for success have more positive attitudes towards doping [17,19]. Cross-sectional research suggests that sport supplement users hold stronger beliefs that doping is effective and are more likely to use doping compared to sport supplement non-users, leading to speculation that supplement users attribute improvements in performance to the supplements themselves [10]. In support of this speculation, studies that measured beliefs about the effectiveness of sport supplements have consistently found that supplement users hold stronger beliefs than non-users [18,20,21,22]. These studies also showed that supplement use was indirectly related to doping attitudes and doping likelihood via sport supplement beliefs [20,21,22]. However, to date, no study has confirmed that this indirect relationship extends to the use of doping substances. The present study therefore tested whether supplement use was related to doping use via supplement beliefs.

Hundreds of different supplements are available to athletes and can be categorized into various types based on their purpose [3,23]. For example, an athlete can take medical supplements to overcome illness and injuries, such as iron and calcium, and take performance supplements, such as creatine and sodium bicarbonate, to improve their performance. The Australian Institute of Sport’s (2019) Sports Supplement Framework [23] distinguishes between groups of sport supplements, which are categorized depending on support for their scientific evidence. Group A supplements have shown strong scientific evidence of their effectiveness in improving sport performance and are separated into sport food supplements (e.g., sports drinks, protein shakes), medical supplements (e.g., iron, calcium), and performance supplements (e.g., caffeine, creatine). Group B supplements require further evidence and include sport supplements such as zinc, amino acids, and carnitine. Group C consists of supplements that have shown no scientific evidence that supported their use for sport performance and includes magnesium, vitamin E, and beta-hydroxy-beta-methyl butyrate. Finally, Group D sport supplements include those that are prohibited or have a high risk of being contaminated with a prohibited substance.

Importantly, while categories of supplements exist, most studies examining the supplement–doping relationship have simply asked participants a single-item question (i.e., yes or no) concerning whether or not they use/used sport supplements [9]. A few studies have adopted a differentiated approach to assess sport supplement use. First, Hildebrandt and colleagues found that the use of steroids by high school students (only 40% engaged in competitive sport) was associated with the use of muscle-building supplements and weight–fat loss supplements but not health–well-being supplements [24]. Moreover, those using muscle-building supplements (but not well-being supplements or weight–fat loss supplements) were more likely to dope due to the belief that the supplements are effective. Second, Hurst and colleagues found that users of sport food/drink supplements (e.g., protein powder), medical supplements (e.g., iron), and ergogenic supplements (e.g., creatine), but not superfood supplements (e.g., goji berries), held stronger sport supplement beliefs than non-users [21]. Moreover, users of medical and ergogenic supplements (but not sport foods and superfoods) also expressed more positive attitudes towards doping than non-users of these supplements. Finally, the stronger pro-doping attitudes expressed by users of sport foods and medical and ergogenic supplements (but not superfoods) were mediated by their stronger beliefs about the effectiveness of sport supplements. Taken together, these findings suggest the possibility that athletes develop beliefs that certain sport supplements are beneficial for performance and these beliefs foster more positive attitudes towards doping in some athletes. Nonetheless, pro-doping attitudes do not necessarily lead to doping behavior [25]. Accordingly, research is needed to assess the role of beliefs in the relationship between supplement use and actual doping behavior. The present study examined this possibility by assessing the use of supplements based on an established framework developed by a major sporting organization.

The aim of our study was to examine the relationship between the use of sport supplements and doping in competitive athletes. We had two specific purposes. The first was to determine the use of sport supplements [23] and doping substances [26] in athletes. We hypothesized a high prevalence of supplement use and a low prevalence of doping use. The second was to examine direct and indirect (via sport supplement beliefs and/or doping likelihood) effects of sport supplement use on doping use. We hypothesized that supplement use and supplement beliefs would be positively associated with doping likelihood and doping use.

## 2. Materials and Methods

### 2.1. Participants

We recruited 345 (194 males, 151 females) athletes, with an average age of 21.83 (SD = 5.32) years, who trained for an average of 8.97 (SD = 7.16) hours per week. They participated in competitive sports (39% individual, 61% team) at various standards (21% international; 15% national, 12% regional, 17% county, 23% club, 12% university). The inclusion criteria were athletes participating in competitive sports and older than 18 years. There were no exclusion criteria. This study was conducted according to the guidelines of the Declaration of Helsinki and approved by the School Ethics Committee of Birmingham University (SPP20211, 2 July 2020).

### 2.2. Sport Supplement Use

The participants were provided with a definition of a sport supplement as “a food, food component, nutrient or non-food compound that is purposefully ingested in addition to the habitually consumed diet with the aim of achieving a specific health and/or performance benefit” [1]. They were then asked a single-item question about whether they used a sport supplement, namely, “In my life as an athlete I have (never used a sport supplement/used a sport supplement” (this single-item question indicated that roughly three out of four athletes (*n* = 263, 76%) reported using a sport supplement). Next, the participants were presented with a list of 23 sport supplements and asked to identify which, if any, they had used in the last six months (Table 1). Their response options were never, previously, less than once per month, monthly, weekly, and daily. Based on the AIS Sport Supplement Framework [23], the list comprised sport food supplements (*n* = 7 items, e.g., sports drinks, electrolytes, sports gels); medical supplements (*n* = 5 items, e.g., iron, calcium, vitamin D), performance supplements (*n* = 6 items, e.g., caffeine, nitrate, glycerol), and other supplements (*n* = 5 items, e.g., zinc, amino acids, antioxidants). We coded the responses as no (never) or yes (previously, less than once per month, monthly, weekly, daily) and computed the sum of the responses (0 = no, 1 = yes) to the 23 supplements as an overall measure of sport supplement use frequency (α = 0.88).

### 2.3. Sport Supplement Beliefs

Beliefs about sport supplements were measured using the Sports Supplements Beliefs Scale [18]. The participants indicated their level of agreement with each of the six items concerning beliefs about the effectiveness of sport supplements (i.e., supplements … improve my performance; are necessary for me to be competitive; improve my confidence; improve my chances of winning; help me realise my potential; improve the quality of my training) on a scale anchored by 1 (strongly disagree) and 6 (strongly agree). The validity and reliability of the scale were established in previous studies (e.g., [18]). The mean of the six ratings yielded a measure of sport supplement beliefs (α = 0.88). Higher scores indicated stronger beliefs in the effectiveness of sport supplements.

### 2.4. Doping Likelihood

Doping likelihood was measured using a hypothetical scenario, adapted from previous doping research [27]. The participants indicated how likely it was that they would use a banned substance in each of nine situations (e.g., financial gain, encouraged by a coach, low chance of detection), on a scale anchored by 1 (not at all likely) and 7 (very likely). The validity and reliability of the scale were established in previous studies (e.g., [27]). The mean of the nine ratings yielded a measure of doping likelihood (α = 0.95). Higher scores indicated greater doping likelihood.

### 2.5. Doping Use

Drug use was measured using a scale from WADA’s Research Package for Anti-Doping Organizations [26]. Participants indicated their use of six classes of performance-enhancing substances and methods (i.e., alphabodies; ephedrine and dimethylamylamine stimulants; anabolic steroids, designer steroids, anabolic agents; erythropoietin and other similar substances; human growth hormone; blood doping methods or blood manipulation) on a response scale with six options (never, previously, less than once per month, monthly, weekly, daily). We coded the responses as no (never) or yes (ever) and computed the sum of the responses (0 = no, 1 = yes) to the six classes of substances and methods as a measure of doping use (α = 0.70).

### 2.6. Procedure

This study employed a cross-sectional survey-based design. Data collection took place during the COVID-19 lockdown between late 2020 and early 2021. Accordingly, the participants were recruited online via social media (e.g., Facebook, Instagram, Twitter). They were sent a link to an anonymous online survey on SmartSurvey, which stores data on an encrypted server. The athletes did not disclose any personal information and were told that all data would be kept anonymous, the information they provided would be used only for research purposes, taking part was voluntary, and honesty in responses was important. After reading the study information sheet and providing informed consent, the athletes completed the measures.

### 2.7. Power and Statistical Analysis

Power calculations using GPower 3.1.5 software [28] indicated that with a sample size of 345, the current study was powered at 0.80 to detect significant (*p* < 0.05) relationships between supplement use, supplement beliefs, doping likelihood, and doping use using Pearson correlation analyses corresponding to a small-to-medium (*r* = 0.15) effect size [29].

The data were analyzed using SPSS version 29 (IBM). The internal consistency (i.e., reliability) of each measure was computed using Cronbach’s (1951) [30] coefficient alpha (α). To examine our first study purpose, we computed the frequencies of using sport supplements, both individually and by category, and computed the frequency of using doping substances. To examine our second study purpose, we computed Pearson correlations to examine the relationships between supplement use, supplement beliefs, doping likelihood, and doping use. We then used PROCESS 4.2 [31] model 6 to determine the direct and indirect (via beliefs and/or likelihood) effects of sport supplement use on doping use. Direct effects are the effects of the predictor on the outcome variable that occur independently of the mediator(s), while indirect effects are the effects of the predictor on the outcome variable via the mediator(s). We used 10,000 bootstrap samples to compute percentile 95% confidence intervals (*CIs*); an effect was significant when the intervals did not cross zero. The Completely Standardized Indirect Effect (CSIE) was reported as the effect size metric, with values of 0.01, 0.09, and 0.25 representing small, medium, and large effect sizes, respectively [29].

## 3. Results

Details of the habitual use of sport supplements are summarized in Table 1. The most commonly used supplements in each category were sports drinks (77%) among sport foods in Group A, multivitamins (55%) among medical supplements in Group A, caffeine (66%) among performance supplements in Group A, and food polyphenols (49%) in Group B. Most athletes (96%, *n* = 332) reported using one or more of the 23 sport supplements. AIS Group A supplements were used by 96% (*n* = 331) of the athletes, which, broken down by category, indicated that 90% (*n* = 310) used sport food supplements, 64% (*n* = 222) used medical supplements, and 72% (*n* = 250) used performance supplements. AIS Group B supplements (i.e., category “other” supplements), were used by 66% (*n* = 227) of the athletes. Furthermore, 11% (*n* = 37) used supplements from one category, 18% (*n* = 61) used two categories, 25% (*n* = 86) used three categories, and 43% (*n* = 148) used four categories of sport supplements.

In terms of the use of doping, very few athletes (4%, *n* = 14) reported using one or more of the six classes of doping substances and methods. Moreover, 14 out of the 14 athletes (100%) who used a doping substance used at least one AIS sport supplement, whereas 318 out of the 331 athletes (96%) who did not use a doping substance used at least one AIS sport supplement.

Table 2 presents the descriptive statistics and correlations among variables. The athletes used many sport supplements, believed supplements were effective for sport performance, were unlikely to use doping substances in hypothetical situations, and used few, if any, doping substances and methods. The Pearson correlations indicated that sport supplement use was positively related to sport supplement beliefs and doping use, sport supplement beliefs were positively related to doping likelihood, and the doping likelihood was positively related to doping use.

**Figure 1:**
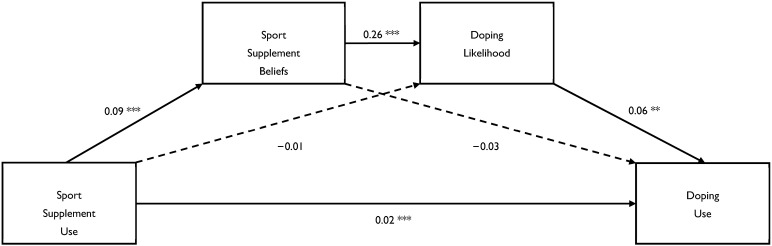
The effects of sport supplement use on doping use, and the mediating roles of sport supplement beliefs and doping likelihood. Note. The values presented are the unstandardized regression coefficients. A solid line represents a significant relationship. ** *p* < 0.01, *** *p* < 0.001.

The PROCESS 4.2 [31] model concerning the direct effect and indirect effects (via beliefs and/or likelihood) of sport supplement use on doping use are depicted in Figure 1. It shows that sport supplement use had a direct effect on doping use, 0.021, 95% *CI* = 0.009, 0.034, *t* = 3.33, *p* < 0.001, and an indirect effect on doping use via supplement beliefs and doping likelihood, CSIE = 0.011, 95% *CI* = 0.003, 0.023, *p* < 0.05. In other words, the increased frequency of doping use was directly explained by the increased frequency of consuming sport supplements and indirectly explained by stronger beliefs about supplements and a greater likelihood of doping in hypothetical situations.

## 4. Discussion

Grounded in the gateway hypothesis, our study sought to determine the prevalence of supplementation and doping, examine the relationship between the use of sport supplements and the use of doping substances, and investigate supplement beliefs and doping likelihood as mechanisms underlying the supplement–doping relationship.

Our first study purpose was to determine the frequency of using sport supplements [23] and doping substances/methods [26]. The list of supplements was taken from Groups A and B of the AIS’s Sports Supplement Framework [23]. Among the 23 sport supplements, prevalence ranged from a low of 7% (bicarbonate) to a high of 77% (sports drink), with an average of 32%. We found that nearly all athletes (96%) used at least one sport supplement in the last six months or previously, and that most athletes used multiple supplements, with athletes consuming seven different classes of supplements on average. Although differences in participants, timeframes, and definitions present difficulties comparing studies, our prevalence rate is consistent with, albeit at the high end of, previous estimates of 40–100% [3]. The current prevalence of supplementation by athletes is similar to a recent study that examined the use of a range of supplements and reported that 97% of athletes used at least one supplement in the last month [7]. Moreover, we found that athletes used a variety of supplements from the three Group A subcategories of supplements that the AIS deems to possess evidence to support their use in sport (sport foods, medical supplements, performance supplements) as well as, albeit to a lesser extent, supplements from the Group B subcategories of supplements, which the AIS stated were currently lacking evidence to support their use by athletes. In contrast, we found that very few athletes reported using one or more of the six classes of doping substances and methods [26]. The prevalence of reported doping in the current sample of athletes (4%) exceeds that typically reported in WADA’s annual anti-doping rule violation database, where the prevalence is less than 1% [32]. However, the current prevalence rate is broadly in line with the majority of past research, with most studies reporting rates of less than 5% [33]. Taken together, and in agreement with past research (e.g., [9]), our findings reveal that most doping users use sport supplements but few sport supplement users also use doping substances.

Our second study purpose was to examine the direct and indirect (via sport supplement beliefs and/or doping likelihood) effects of sport supplement use on doping use. Extending past research [20,21,22], we found that sport supplement use was indirectly related to doping use via sport supplement beliefs and doping likelihood (Figure 1). These replications implicate beliefs as an intermediary factor in supplement–doping relationships and suggest that the perceived performance benefits of using chemically active substances may permit athletes to view the use of doping substances as similarly effective, which may foster positive attitudes towards their use. Moreover, we found that sport supplement use was indirectly related to doping use via a combination of both sport supplement beliefs and doping likelihood, suggesting that athletes who use sport supplements are more likely to use doping substances when they believe that the consumption of sport supplements is likely to enhance their performance and when they are tempted to use prohibited substances in specific situations that they might encounter, such as those associated with reduced personal responsibility and increased rewards. Accordingly, both of these personal factors (i.e., beliefs and likelihood of being tempted) may increase an athlete’s risk of using doping substances.

In sum, our findings broadly replicate previous research [20,21,22,24], and, at least at first glance, partially support the gateway hypothesis (cf. [15]). Specifically, athletes who used more sport supplements were more likely to use more doping substances and methods. As specified by the gateway hypothesis, athletes’ consumption of permitted substances represents the opening of a gate to the consumption of prohibited substances. Despite all doping users also being supplement users, most supplement users were not users of doping substances. It could therefore be argued that the current data fail to offer clear support for the gateway hypothesis. This reasoning underscores the weaknesses associated with using a simple binary yes/no question about supplement use that is most commonly used in previous research [9]) and instead emphasizes the benefits of assessing supplement use in more detail, such as the total number of supplements used. Finally, the current findings argue for the need to look for potential moderators of the supplement–doping relationship, such as the demands of the sport, time of the season, and motivation.

## 5. Limitations

The current project yielded some novel and important evidence concerning sport supplements and doping. However, our findings should be interpreted in light of potential limitations. First, the current study is the first, to our knowledge, to use the AIS framework [23] to assess the use of sport supplements in this context. Although our findings broadly replicated those reported by studies using a similar albeit less detailed framework [21], they need to be replicated and extended using other frameworks. Indeed, other sport organizations offer guidance on supplements that could be examined in future research. Second, for simplicity, we focused on describing the models concerning the overall use of supplements; however, we found similar effects when we examined the use of supplements broken down into four subcategories of supplements. (Separate mediational analyses involving each of the four categories of sport supplement use (i.e., sport foods, medical supplements, performance supplements, other supplements) as the predictor variable yielded similar direct and indirect effects as those reported using the total number of sport supplements as the predictor variable. The only discrepancy was that the direct effect was not significant for the sport foods category.) It is notable that the prevalence of supplementation was considerably higher using the detailed 23-item framework (96%) compared to the standard one-item question (76%). This discrepancy suggests that some athletes lacked an understanding of what constitutes a sports supplement, and implies that past studies may have underestimated supplement use. Third, the data were collected during the COVID-19 lockdown. Accordingly, the findings need to be replicated now that athletes are no longer restricted. Fourth, the supplement and doping measures were self-reported. Future studies could also include biochemical analysis. Fifth, the study design was cross-sectional. Future studies could consider longitudinal designs to track changes in usage and beliefs over time. Finally, the proportion and number of doping users were small, and, therefore, the interpretation of the model (Figure 1) describing the effects of supplement use on doping use should be interpreted with caution before it is confirmed by other research studies.

## 6. Conclusions

We documented the frequent and widespread use of sport supplements across multiple categories according to the Australian Institute of Sport’s (2019) Sports Supplement Framework [23]. This is the first study to use this well-established framework for categorizing supplement use. We also noted that our heterogenous sample of athletes, competing at various levels along the sport pathway, rarely used some of the most commonly reported performance-enhancing substances and methods that have been banned by [34]. This suggests that athletes may seek to meet their needs for performance enhancement via sport supplementation and that most athletes’ needs are being satisfied safely and sufficiently within the rules of sport. However, these contrasting prevalence rates, namely, 96% supplement use versus 4% doping use, describe a funneling effect, suggesting that only a small fraction of supplement users progress to become doping users. To help understand this effect, which on its own represents a low risk for doping, we identified two factors, namely, beliefs about the efficacy of sport supplements for performance and the likelihood of doping in tempting situations, that may help identify athletes who are at increased risk of doping. Our findings may be used by sport organizations to identify athletes who may be considered to be at increased risk for doping so that they can be targeted for anti-doping monitoring, education, and training.

## Figures and Tables

**Table 1:** Use of sport supplements based on AIS classifications expressed as a percentage of the total sample (*n* = 345). Zinc was classified in Group B when these data were collected.

Group	Subcategory	Item	Never	Previously	<1/Month	Monthly	Weekly	Daily
A	Sport foods	sports drink	23	6	24	18	23	7
A	Sport foods	sports gel	62	8	17	6	6	1
A	Sport foods	sports confectionery	68	3	16	7	5	1
A	Sport foods	sports bar	41	6	22	17	13	4
A	Sport foods	electrolyte supplement	53	6	19	9	10	4
A	Sport foods	isolated protein supplement	62	4	7	5	10	12
A	Sport foods	mixed macronutrient supplement	81	2	10	2	4	2
A	Medical supplement	iron supplement	72	4	6	2	5	11
A	Medical supplement	calcium supplement	82	2	6	1	2	7
A	Medical supplement	multivitamin supplement	45	5	10	5	4	30
A	Medical supplement	vitamin D supplement	59	4	8	4	5	20
A	Medical supplement	probiotics	81	2	7	2	3	5
A	Performance supplement	caffeine	34	1	9	4	15	36
A	Performance supplement	B-alanine	92	0	4	1	2	1
A	Performance supplement	bicarbonate	93	1	4	1	1	0
A	Performance supplement	beetroot juice/nitrate	92	1	3	1	2	1
A	Performance supplement	creatine	80	6	5	2	1	6
A	Performance supplement	glycerol	91	1	5	1	1	0
B	Food polyphenols	food polyphenols [e.g., berries, currants]	51	0	6	8	23	11
B	Metabolism compounds	metabolism compounds [e.g., carnitine, phosphate]	73	4	5	3	8	8
B	Zinc	zinc	79	2	5	4	3	7
B	Amino acids	amino acids [e.g., BCAA/leucine, tyrosine]	82	2	5	3	4	4
B	Antioxidants	antioxidants [e.g., vitamin C and E]	61	2	7	7	9	14

**Table 2:** Descriptive statistics (range) and zero-order correlations. ** *p* < 0.01, *** *p* < 0.001.

Variable	*M*	*SD*	1	2	3
1. Sport supplement use (0–23)	7.44	5.06			
2. Sport supplement beliefs (1–6)	3.12	1.11	0.40 ***		
3. Doping likelihood (1–7)	2.47	1.46	0.05	0.18 ***	
4. Doping use (0–6)	0.08	0.56	0.18 ***	0.05	0.14 **

## Data Availability

Data are available from the corresponding author.
